# Vascular reactivity in post-COVID-19 patients: analysis and correlation with functional capacity

**DOI:** 10.36416/1806-3756/e20250268

**Published:** 2025-11-14

**Authors:** Luara Inocêncio Pereira Silva, Mônica Corso Pereira, Rickson Coelho Mesquita, Bruna Scharlack Vian, Ligia dos Santos Roceto Ratti

**Affiliations:** 1. Faculdade de Ciências Médicas, Universidade Estadual de Campinas - UNICAMP - Campinas (SP) Brasil.; 2. Departamento de Clínica Médica, Faculdade de Ciências Médicas, Universidade Estadual de Campinas - UNICAMP - Campinas (SP) Brasil.; 3. Instituto de Física Gleb Wataghin, Universidade Estadual de Campinas - UNICAMP - Campinas (SP) Brasil.; 4. Hospital de Clínicas, Universidade Estadual de Campinas - UNICAMP - Campinas (SP) Brasil.

## TO THE EDITOR:

This article discusses a technique for assessing vascular reactivity in post-COVID-19 patients, whose outcomes 4 months after hospital discharge have yet to be fully understood. Understanding vascular reactivity in such patients may help elucidate the relationship of COVID-19 sequelae, prognosis, and long-term functionality. 

During the COVID-19 pandemic, critically ill patients frequently required ICU admission, oxygen therapy, and mechanical ventilation, often resulting in complications related to comorbidities and prolonged hospitalization. Vascular alterations, chronic/acute inflammation, and worsening of preexisting conditions have been associated with poor outcomes.^1^ ^,^ ^2^ Patients presenting with any of the aforementioned conditions commonly experience functional decline, muscle loss, ICU-acquired weakness, and reduced independence.^3^ In patients with COVID-19, complications such as microthrombi, thromboembolic events, epithelial damage, and elevated proinflammatory cytokines are prevalent, and the dysregulated inflammatory response can lead to vasculitis, coagulation disorders, and impaired gas exchange caused by altered capillary blood flow.^4^ Evidence suggests that vascular dysfunction as assessed by near-infrared spectroscopy (NIRS) and vascular occlusion testing (VOT) is associated with endothelial damage, more severe ARDS, and increased mortality.^5^ When associated with submaximal tests such as the six-minute walk test (6MWT), NIRS can aid in assessing musculoskeletal, vascular, and pulmonary responses in post-COVID-19 patients, revealing potential associations between cardiovascular and microvascular dysfunction.^6^ We sought to evaluate vascular reactivity in post-COVID-19 patients using VOT and to explore the correlation of VOT with the 6MWT and handgrip strength (HGS). This was a prospective observational study conducted at the outpatient clinic of the *Universidade Estadual de Campinas Hospital de Clínicas*, located in the city of Campinas, Brazil. The study was approved by the local research ethics committee (Protocol no. CAAE 34454920.7.0000.5404). Between February and December of 2022, individuals > 18 years of age discharged after RT-PCR-confirmed COVID-19 pneumonia and referred to the post-COVID-19 outpatient clinic were invited by telephone to undergo functional and vascular assessments at our physical therapy and occupational therapy service. Exclusion criteria included upper limb injuries preventing NIRS sensor placement, inability to perform the 6MWT, and cognitive impairment interfering with test understanding. Each participant underwent a single evaluation session including the following: vascular reactivity assessment via NIRS and VOT; HGS testing with a hydraulic dynamometer (Saehan Corporation, Changwon, South Korea); and the 6MWT, performed in a 30-m corridor in accordance with the American Thoracic Society guidelines.^7^ The six-minute walk distance (6MWD) was compared with reference equations for the 6MWT in healthy individuals in Brazil.^7^

Before VOT, body fat was measured using an adipometer (Slim Fit; Balmak, Santa Barbara d’Oeste, Brazil) on the dominant arm. Blood pressure was measured with a sphygmomanometer on the contralateral arm (AccuMed, Rio de Janeiro, Brazil). The NIRS sensor (PortaMon; Artinis Medical Systems, Elst, the Netherlands) was secured to the opposite forearm with a black band to reduce ambient light interference. After resting in the supine position for 5 min, a cuff was inflated to 50 mmHg above systolic pressure and maintained for 3 min. A pulse oximeter (G-TECH, Belo Horizonte, Brazil) was placed on the contralateral arm to monitor HR and SpO_2_. NIRS data collection continued for 5 min after occlusion, totaling approximately 13 min. The parameters analyzed included desaturation slope, resting saturation, minimum saturation, maximum saturation, resaturation slope, and AUC. Data were transferred via Bluetooth^®^ to a notebook (Lenovo, Beijing, China) and analyzed using DOS-based software. 

COVID-19 has systemic repercussions that have yet to be fully understood, especially among hospitalized individuals. We observed that hospital stays longer than 10 days can have a negative impact on resaturation slope values as measured via NIRS during VOT, even in the absence of clearly impaired HGS or 6MWT performance approximately 4 months after discharge. 

Of a total of 88 post-COVID-19 patients who had been admitted to our hospital, 47 completed the evaluation protocol (Table 1). All of the study participants achieved more than 80% of the predicted 6MWD (mean, 476.3 ± 98.3 m), and mean HGS was 28.8 ± 11.3 kgf. Mean forearm skinfold thickness was 6.0 ± 10.8 mm. Of the NIRS variables, only the resaturation slope showed a significant correlation with clinical parameters (r = −0.34; p = 0.03). No significant correlations were found between hospital length of stay or time since hospital discharge and vascular or functional outcomes (p > 0.05). 

**Table 1:** General characteristics of the study sample.^a^

Characteristic	Post-COVID-19 (N = 47)
Sex, female/male	12/35
Age, years	56 ± 1.5
Length of hospital stay, days	
1-5	14 (29.8)
6-10	11 (23.4)
> 10	22 (46.8)
Time since hospital discharge, days	139.6 ± 57.6
Use of corticosteroids, %	44.7
IMV/NIV, %	27.7/4.2
HFNC, %	4.3
Comorbidities	
Hypertension, %	48.9
Smoking, %	34.0
Diabetes, %	29.8
Obesity, %	10.6
6MWD, m	476.3 ± 98.3

IMV: invasive mechanical ventilation; NIV: noninvasive ventilation; HFNC: high-flow nasal cannula; and 6MWD: six-minute walk distance. ^a^Values expressed as n/n, n (%), or mean ± SD, except where otherwise indicated.

VOT showed moderate correlations with the 6MWT: a negative correlation between AUC and resting HR (r = −0.42; p = 0.001); a positive correlation between AUC and the 6MWD (r = 0.39; p = 0.01); and a weak but significant positive correlation between the resaturation slope and resting SpO_2_ (r = 0.37; p = 0.01). When patients were stratified by corticosteroid use during hospitalization, significantly lower maximum saturation values (71.74 ± 3.52%) and lower post-6MWT SpO_2_ (95.65 ± 2.69%) were observed in the corticosteroid group (p = 0.03 and p = 0.04, respectively), suggesting a possible impact on microvascular recovery. However, the use of invasive mechanical ventilation did not significantly affect NIRS outcomes. 

**Figure 1:**
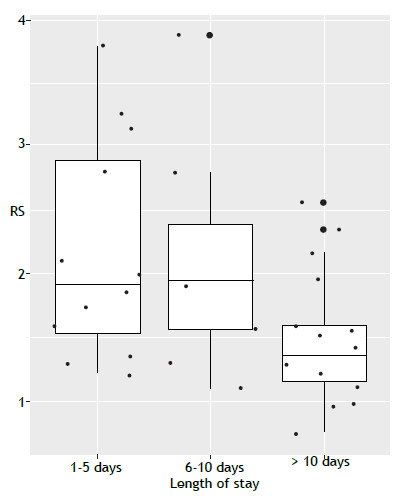
Dispersion of resaturation slope (RS) values, by length of hospital stay.

Patients who had been hospitalized for more than 10 days had significantly lower resaturation slope values (1.47 ± 0.51 %/s) than those who had been hospitalized for 1-5 days or 6-10 days (p = 0.02), reinforcing the hypothesis that prolonged hospitalization is associated with impaired microvascular reactivity (Figure 1). Although the functional consequences were not evident in the short term, these findings may indicate long-term risks related to endothelial dysfunction.^8^ ^,^ ^9^ The need for further prospective studies remains critical to clarify the clinical implications of these microcirculatory alterations. 

COVID-19 is known to cause significant endothelial dysfunction associated with vasoplegia, systemic inflammation, and prothrombotic responses.^8^ Previous studies have demonstrated that microcirculatory changes identified via NIRS may correlate with ARDS severity and poor prognosis.^8^ In our analysis, lower resaturation slope values were observed in patients who received corticosteroids during hospitalization, suggesting that microcirculatory recovery may be affected by such interventions.^9^ Although a reduced resaturation slope had no direct impact on functional performance, it may represent an early marker of endothelial dysfunction and increased risk for long-term complications.^8^

Although there was no significant correlation between invasive mechanical ventilation and NIRS parameters, hospital length of stay showed a significant association. This factor has been widely linked to peripheral and diaphragmatic muscle loss and higher mortality rates.^10^ Even patients with preserved submaximal exercise responses have shown altered vascular reactivity,^10^ emphasizing the relevance of these findings as potential clinical predictors. Despite the lack of randomized clinical trials explaining why endothelial dysfunction as detected by NIRS and VOT does not always translate to reduced function, this condition may still signal an increased risk of organ dysfunction and mortality. 

It is of note that the patients evaluated in the present study were not part of the first wave of the COVID-19 pandemic (from 2020 to early 2021), when vaccination was limited and clinical management protocols were still evolving. Our patients likely benefited from improved care, wider vaccine coverage, refined therapeutic strategies, and greater experience from multidisciplinary teams.^3^ Finally, the limitations of the present study include loss to follow-up as a result of outdated contact information, relocation, death, or transportation issues, all of which reduced the final sample size. In addition, external factors such as ambient light, poor sensor fixation, and involuntary movements may have compromised the quality of the NIRS signal. 

## References

[1] Yaqub MA, Wieringa FP, van der Steen AFW, Heger M (2024). Non-invasive monitoring of microvascular oxygenation and reactive hyperemia using hybrid, near-infrared diffuse optical spectroscopy for critical care. J Vis Exp.

[2] Mehta P, McAuley DF, Brown M, Sanchez E, Tattersall RS, Manson JJ (2020). COVID-19 consider cytokine storm syndromes and immunosuppression. Lancet.

[3] Jimeno-Almazán A, Pallarés JG, Buendía-Romero Á, Martínez-Cava A, Franco-López F, Sánchez-Alcaraz Martínez BJ (2021). Post-COVID-19 syndrome and the potential benefits of exercise. Int J Environ Res Public Health.

[4] Teuwen LA, Geldhof V, Pasut A, Carmeliet P (2020). COVID-19 the vasculature unleashed. Nat Rev Immunol.

[5] Mesquida J, Espasa A, Hermida C, Saludes P, Gimeno A, Betbese AJ (2021). Peripheral microcirculatory alterations are associated with the severity of acute respiratory distress syndrome in COVID-19 patients admitted to intermediate respiratory and intensive care units. Crit Care.

[6] Bec KB, Grabska J, Huck CW (2020). Near-infrared spectroscopy in bio-applications. Molecules.

[7] Iwama AM, Andrade GN, Shima P, Tanni SE, Godoy I, Dourado VZ (2009). The six-minute walk test and body weight-walk distance product in healthy Brazilian subjects. Braz J Med Biol Res.

[8] Perico L, Benigni A, Casiraghi F, Ng LFP, Renia L, Remuzzi G (2021). Immunity, endothelial injury and complement-induced coagulopathy in COVID-19. Nat Rev Nephrol.

[9] Cruz DA, Gava GC, Nascimento LO, Alves DR, Cardoso AC, Avelar NP (2021). Impacts of invasive mechanical ventilation on patients from COVID-19 integrative review [Article in Portuguese]. Res Soc. Dev.

[10] Roberts HC, Denison HJ, Martin HJ, Patel HP, Syddall H, Cooper C (2011). A review of the measurement of grip strength in clinical and epidemiological studies towards a standardised approach. Age Ageing.

